# The Human Cell Atlas: Promises, recent developments, and bridging the African single-cell data gap

**DOI:** 10.4102/ajlm.v13i1.2583

**Published:** 2024-12-19

**Authors:** Enahoro S. Abhulimen

**Affiliations:** 1 College of Medicine, University of Ibadan, Ibadan, Nigeria

## Introduction

The Human Genome Project changed everything. The 13-year-long campaign to create a reference human genome finally ended with the announcement of an essentially complete draft of the sequence in 2003. Advances in DNA sequencing technology, such as the development of automated Sanger sequencing machines like the ABI PRISM range manufactured by Applied Biosystems, paved the way for the realisation of the mega project 2 years ahead of schedule.^1^ The creation of ambitious initiatives such as this is constrained by available technology; therefore, evolving genomic technologies have led to the development of several other large-scale genomics initiatives.

## Conception and development

The Human Genome Project produced a reference genome sequence, which is identical in nearly all of the 37 trillion cells in the body. However, there is a wide diversity of cell types in the body, which arises from differences in the activated genes within these cell phenotypes. Recent revolutions in genomics, such as developments in single-cell RNA sequencing (scRNA-seq) technologies, for example the introduction of in situ barcoding, have enabled the evaluation of gene expression at cellular resolution for hundreds of thousands of cells in parallel.^2^ Additionally, advancements in spatial transcriptomics methods now allow for the mapping of gene expression, while retaining spatial information about the distribution of cells within tissue samples.^2^ Coupled with modern computational biology methods necessary for analysing this data, these developments have made it possible to create a reference atlas of all the cell types in the human body, which would mark the completion of the 150-year-old effort to categorise these cell types. These advances have led to the conception of a human cell atlas, as they made realising the mammoth undertaking both feasible and economically viable.

Therefore, in 2016, Sarah Teichmann (Wellcome Sanger Institute, Cambridge, United Kingdom) and Aviv Regev (Broad Institute, Massachusetts Institute of Technology, Cambridge, Massachusetts, United States) co-founded the Human Cell Atlas (HCA), an open, large-scale, scientist-led consortium aimed at mapping out in detail the cellular architecture of the human body.^2,3^ Global researchers convened for an inaugural planning meeting in London in 2016, and since then, membership of the consortium has grown to more than 3000 members from over 99 countries, spread across 18 biological networks.^3^ These biological networks were created for organisational efficiency, with each network composed of scientific experts in various systems and organs, working together from around the world to map out these systems. According to the HCA’s white paper,^4^ the first draft of the atlas is expected to contain 30–100 million cells from all major human organs and systems. The plan to structure this ambitious project into shorter-term, achievable intermediate goals reflects insights from the past, mirroring the approach adopted during the Human Genome Project, which led to the completion of the project years ahead of expected projections.^5^ The consortium has now mapped tens of millions of individual cells across the 18 biological networks, encompassing male and female individuals of varied ethnic backgrounds, and published over 190 scientific papers revealing new insights into human biology and disease.^3^ The data from these networks are stored in the HCA Data Portal,^6^ a publicly accessible cloud-based platform, enabling scientists from around the world to access all the collected single-cell data. The complete atlas is expected to map at least 10 billion cells^4^ across all human tissues, organs, and systems, including developmental cells and cells from individuals with various diseases, with the projected time for its completion spanning another estimated 5–10 years.

## Promises and prospects

The HCA promises to transform our understanding of human biology, thereby revolutionising how we treat diseases. The applications of a completed atlas range from advancements in our diagnostic capabilities to understanding drug efficacy and toxicity, as well as facilitating drug target discovery and advancements in regenerative medicine. A proposed diagnostic application of the atlas is the creation of essentially a ‘complete blood count (CBC) 2.0’, which is a more complex characterisation of the blood cells in the routine test. This involves genomic profiling of the various blood cell components giving a better understanding of the experiences the cells have undergone and their associations with various diseases, such as autoimmune diseases and malignancies.^4,7^ For histology, this technology aims to transform primitive haematoxylin and eosin (H&E) staining by integrating genomic profiling and spatial technology, thereby creating an ‘H&E 2.0’ approach that offers a molecular view of tissues.^7^ Another promise of the complete atlas is improved knowledge of why drugs may or may not be functional or predict off-target locations where they may express their effects, by studying gene expression similarities between healthy and diseased cells. Also, its application in regenerative medicine provides the opportunity to create precise organoid models and in vitro cells, which could enable targeted cell therapies.^4^ Eukaryotic cells are already being synthesised, such as in the Synthetic Yeast Genome Project (Sc2.0),^8^ indicating the potential for the creation of synthetic human cells that could be used to study disease models or for therapeutic purposes. The HCA data could therefore be leveraged to design more accurate human cells, thereby revolutionising the emerging field of synthetic genomics. These promises may seem ambitious, but the prospect of even a few of these innovations materialising would create a paradigm shift, effectively transforming healthcare as we know it.

Notwithstanding, one would have to consider the practicability of these potential applications of the atlas data. For example, the promise of a ‘CBC 2.0’ and ‘H&E 2.0’ approach would be exciting to a researcher, but full adoption of these technologies may take several years, as the current technology is still adequately functional, and there might be clinician hesitation to pivot from existing reliable technology. Also, the affordability or availability of the required technology to carry out these new tests in low- and middle-income countries, where there is still limited access to current technology, are other factors that may limit widespread adoption. Furthermore, concerning the applications of the HCA in synthetic genomics, the HCA data may not be directly responsible for the revolution in currently available technology as improvements in our genetic engineering capabilities are still required to create major advancements in this field.^7^ However, as the era of personalised medicine approaches, the atlas could be used to make the therapeutic applications of synthetic genomics more precise.

## COVID-19: A novel challenge

In 2019, the emergence of the coronavirus disease 2019 (COVID-19) pandemic allowed for new and unprecedented applications of the HCA data. The lung biological network, which is focused on creating a complete atlas of the human lung and, eventually, the broader HCA community, mobilised efforts to tackle this disease, subsequently resulting in the creation of a COVID-19 cell atlas. Initially, efforts were aimed at using the available HCA data to understand the underlying biology of this disease, with subsequent studies using data from samples of COVID-19 patients. Studies from the consortium compared atlas data from healthy tissues with data from samples taken from postmortem tissue of patients with the disease, leading to the discovery of viral targets, and isolation of genes related to severe COVID-19 infections.^7^ The ACE2 receptors and TMPRSS2 protease were found to be the major receptors facilitating viral entry. Research showed that the ACE2 receptor binding affinity was found to influence viral replication rates and the severity of the disease.^9^ Thus, expression of the *ACE2* and *TMPRSS2* genes across various tissues was investigated using scRNA-seq data sets from the HCA. Both genes were expressed in a wide variety of tissues, with the *TMPRSS2* gene having a wider distribution among the tissues sampled, suggesting that the expression of the *ACE2* gene was a rate-limiting factor for viral infection. The study also found the highest expression rate of these genes in nasal ciliated and goblet cells.^9^

These findings provided insight into the most likely sites of entry and reservoir for the virus, a discovery that was crucial in shaping mask-wearing policy by helping us understand the routes of viral transmission in the early days of the pandemic. Furthermore, analysis of scRNA-seq data of immune cells in individuals who received the COVID-19 vaccines helped researchers to better analyse the efficacy of these new vaccines.^7^ The adaptability displayed by the HCA community, alongside the wide range of practical implementations of the atlas data during the pandemic, illustrates the potential for the diverse applications of the HCA. This is especially impressive considering that the initiative was barely 3 years old at the start of the pandemic, leading us to wonder about the possible capabilities of a completed atlas.

## Recent developments

Several studies published by the HCA have transformed our understanding of human biology and disease. For instance, the human development biological network used scRNA-seq and spatial transcriptomics to conduct a spatial analysis of gene expression patterns in human limb development across different gestational ages. This study^10^ has corrected previously conceived expectations about the pattern of growth of the limb digits, and also highlighted distinct anatomical separation between genes associated with brachydactyly and polysyndactyly, providing new insight into the development of these conditions. The various biological networks of the HCA have also been involved in creating roadmaps that outline plans to ensure diversity in the samples collected, identify potential obstacles, and establish goals for creating a draft atlas of their respective systems. A few of these roadmaps have already been published for the skin, gut, lung, human development, and oral and craniofacial cell atlas.

With the HCA data sets rapidly expanding, these networks have pivoted to creating integrated atlases, such as the development of the first integrated single-cell atlas of the human lung. The integrated Human Lung Cell Atlas consists of 2.4 million cells from 49 data sets consisting of healthy lung data and data from more than 10 lung diseases, which were re-annotated to create a consistent cell type reference.^11^ The results obtained from merging these data sets have already begun to expand our understanding of various lung disease states. For example, the discovery of similar gene activity in profibrotic SPP1^+^ monocyte-derived macrophages in lung carcinoma, COVID-19, and idiopathic pulmonary fibrosis has led to the discovery of potential therapeutic targets for these conditions.^11^ The development of integrated atlases by the different biological networks will represent a defining milestone in the consortium’s effort, as the eventual goal is the creation of essentially an integrated atlas of the whole human body. Therefore, it is expected that we will see several more of these atlas integrations in the near future.

## The African ‘single-cell data gap’

The HCA is similar to the Human Genome Project in many ways, as they are both ambitious large-scale initiatives, with the latter inspiring the conception of the former.^5^ However, a major difference between the two initiatives is that while the goal of the Human Genome Project was to create a single DNA sequence, the HCA aims to create a data set of diverse cell types. This necessitates adequate representation in terms of age, gender, ethnicity, geographical location, and disease states. Regardless, 65% of the data from the integrated Human Lung Cell Atlas belonged to individuals of European ethnicity,^11^ indicating a need for increased representation in the biospecimens obtained by the consortium. There is already a general underrepresentation of minorities in single-cell data and the HCA may further widen this disparity, leading to comparatively worse healthcare outcomes within these populations.^12^ Overcoming this challenge will therefore be an important determinant of the HCA’s eventual success.

The disproportionate representation of minority populations, such as Africans, in the HCA data seems to follow a similar trend observed in most current large-scale genomic studies. For example, African data constituted 1.1% of the world’s genome-wide association studies data in 2021, despite Africa making up about 17% of the world’s population.^13^ The HCA’s Equity Working Group has devised strategies to ensure equity in its efforts, such as increasing global participation and collaboration in the initiative, especially in low- and middle-income countries, where adequate knowledge about or facilities for scRNA-seq might be scarce.^14^ Therefore, efforts must be made to prevent further widening of, or to bridge, any existing ‘single-cell data gaps’ that may have developed due to a lack of participation by African institutions. Several organisations, such as The African Centre of Excellence for Genomics of Infectious Diseases, 54Gene, Inqaba Biotec, and The Human Heredity and Health in Africa Consortium, are already working to bridge Africa’s genomic data gap, and could be partnered with to advance the HCA on the continent. Another potential approach could involve prioritising obtaining samples from local ethnic minorities by member institutes, while also making efforts to secure both local and international funding for infrastructure development in low- and middle-income countries, such as those within the African continent.

## Predictions and outlook

Nevertheless, from the author’s perspective, the initiative has already started to deliver on its promises, considering the numerous insights it has made into our understanding of human biology^10,11^ and diseases,^7,9,11^ even though it is still at least 5–10 years from its completion. However, some of the initial promises of the atlas, such as a ‘CBC 2.0’ or ‘H&E 2.0’, may require several years for development and widespread adoption. Therefore, it is anticipated that the true promise of the atlas lies in its adaptability and all the unforeseen, practical ways in which we will come to use it, just as we did during the COVID-19 pandemic. It is also expected that over time, as genomic technologies continue to advance, the initiative may evolve to map cells with even greater resolution while observing interactions between individual cells at the genomic level. This could involve the use of multi-omics-based technologies to observe heterogeneity among cell populations we currently believe to be homogeneous, further enhancing the potential of the completed atlas.
